# Investigation of Phase Transformation and Fracture Pattern as a Result of Long-Term Chewing Simulation and Static Loading of Reduced-Diameter Zirconia Implants

**DOI:** 10.3390/ma17194719

**Published:** 2024-09-26

**Authors:** Pelin Atalay Seçkiner, Fehmi Gönüldaş, Bora Akat, Arda Buyuksungur, Kaan Orhan

**Affiliations:** 1 Department of Prosthodontics, Faculty of Dentistry, Niğde Ömer Halisdemir University, Niğde 51240, Turkey; 2 Department of Prosthodontics, Faculty of Dentistry, Ankara University, Ankara 06100, Turkey; dt.fehmi@gmail.com (F.G.); boraakat@gmail.com (B.A.); 3 Department of Basic Medical Sciences, Ankara University, Ankara 06100, Turkey; abuyuksungur@ankara.edu.tr; 4 Department of Oral and Maxillofacial Radiology, Faculty of Dentistry, Ankara University, Ankara 06100, Turkey; knorhan@dentistry.ankara.edu.tr; 5 Department of Oral Diagnostics, Faculty of Dentistry, Semmelweis University, 1088 Budapest, Hungary

**Keywords:** zirconia implant, chewing simulation, phase transformation, fracture pattern

## Abstract

While zirconia implants exhibit osseointegration comparable to that of titanium, concerns arise regarding low-temperature degradation and its potential impact on fracture strength. This study investigated the phase transformation and fracture characteristics of zirconia dental implants after aging through chewing simulation and subsequent static loading. The experimental setup involved 48 one-piece monobloc zirconia implants with diameters of 3.0 mm and 3.7 mm that had straight or angled abutments, with crown restorations, which were divided into six groups based on intraoral regions. The specimens underwent chewing simulation equal to five years of oral service, which was followed by static loading. Statistical analyses were performed for the data obtained from the tests. After dynamic and static loadings, the fractured samples were investigated by Raman spectroscopy to analyze the phase composition and micro-CT to evaluate fracture surfaces and volume changes. According to the results, narrow-diameter zirconia implants have low mechanical durability. The fracture levels, fracture patterns, total porosity, and implant fracture volume values varied according to the implant diameter and phase transformation grade. It was concluded that phase transformation initially guides the propagation of microcracks in zirconia implants, enhancing fracture toughness up to a specific threshold; however, beyond that point, it leads to destructive consequences.

## 1. Introduction

Aluminum oxide ceramic implants were generated and examined during the same period as titanium alloys. However, they were not successful as an implant material option because of their lower mechanical resistance; additionally, the hardness of alumina ceramics has a greater potential to cause bone resorption [1,2,3]. Currently, 3 mol% yttria tetragonal zirconia polycrystalline (3Y-TZP) is recognized as a hard, wear-resistant, and osteoconductive material appropriate for stress-handling implant applications. Animal and clinical studies have shown that zirconia implants exhibit osseointegration comparable to that of titanium implants, superior biocompatibility regarding reduced plaque accumulation and inflammation, and higher affinity for osteoblasts and soft tissues [4,5,6,7,8,9]. With increasing aesthetic demands in dentistry, the research and development interest in and market share of ceramic implants have recently been renewed [10,11]. A considerable variety of zirconia implants are available today, the effectiveness of which have been proven in animal studies [9,11]. Nevertheless, long-term clinical trials to confirm clinical success are still needed [7].

Among the various types of zirconia ceramics, 3Y-TZP, which exhibits appropriate flexural strength and fracture toughness values due to its phase-transformation toughening mechanism, is presently assumed as the standard combination for ceramic dental implant manufacture [12]. However, the tetragonal–monoclinic (t-m) phase transformation of Y-TZP progresses spontaneously in moist conditions like those in the oral cavity with roughness and microcracks in the zirconia material being the probable outcome. Mechanical stress, thermal aging, and chemical influences may lead to low-temperature degradation (LTD), which reduces the fracture strength of zirconia implants and leads to premature ceramic fracture [13,14,15,16].

Long-term clinical data are still lacking [17]; therefore, it is essential to conduct preclinical laboratory studies that replicate the oral environment to predict the long-term clinical behavior and failure of new implant materials. To assess the fatigue strength of materials, clinical conditions can be simulated in vitro using a chewing simulation device [18,19], and a static loading test can then be applied to evaluate the fracture strength [19,20]. T-m transformation as a result of LTD can be evaluated using Raman spectroscopy. Zirconia’s strong Raman signal in its t- and m-variants allows for the determination of the amount of m-zirconia relative to the total zirconia due to their distinct vibrational spectra [21]. A variety of specimens, such as mineralized tissues like teeth and bones, ceramics, polymers, and biomaterial scaffolds, can be analyzed using micro-CT [22,23]. The micro-CT system allows for projections rotated along multiple imaging approaches to create 3D-reconstructed images of specimens, observations of their surface areas, and calculations of their volume changes [24].

This study aimed to investigate the phase transformation of zirconia implants subjected to aging by a chewing simulation, followed by static loading until failure, using Raman spectroscopy and micro-CT to analyze the fracture surface.

## 2. Materials/Methods

### 2.1. Experimental Setup

A total of forty-eight monobloc zirconia implants and crown restorations, made from monolithic zirconia, were utilized. The samples were divided into six groups according to three different intraoral regions and abutment angulation: Group 11-S (upper central incisor with straight abutment), Group 11-A (upper central incisor with angled abutment), Group 31-S (lower central incisor with straight abutment), Group 31-A (lower central incisor with angled abutment), Group 13-S (upper canine with straight abutment), and Group 13-A (upper canine with angled abutment). Details regarding the samples are presented in Table 1. The sample size of each group was determined based on the effect size of a previous study, with effect size = 0.80, α = 0.5, and required power = 0.93 [25].

**Figure 1:**
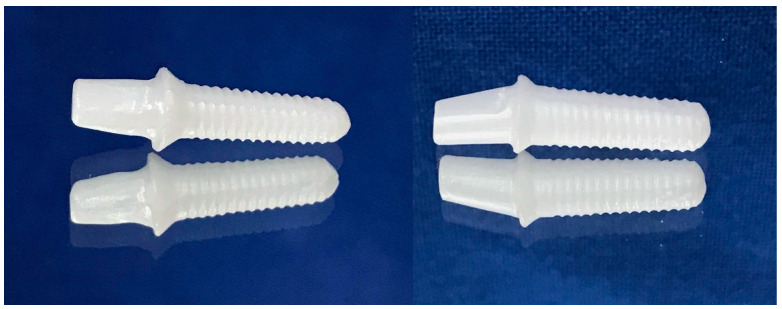
Monoblock zirconia implants with straight and 15 degrees angled abutment designed chamfer edge with 1 mm of width and gingiva height. The implant bodies were conical, the taper angle was 3 degrees, and the screw threads had an angle of 10 degrees, a depth of 0.35 mm, and a thread pitch of 1.8 mm.

All samples were milled from pre-sintered 3Y-TZP blocks (Upcera, Pforzheim, Germany) (Figure 1) using CAD/CAM technology (Yenadent D15 CNC milling device, Yenadent Dış. Tic. Ltd., İstanbul, Turkey). The specimens were subjected to a sintering process in a specialized sintering furnace (ELV MoS Protherm Furnaces, Nabertherm GmbH, Lilienthal, Germany) considering the manufacturer’s suggestion by performing heat at 1500 °C under high pressure in an inert atmosphere (High Isostatic Pressing-HIP) (SetDent Dental Labor, Kayseri, Turkey). All of the samples were cleaned using ethanol for five minutes through ultrasonic cleaning (BioSonic UCI-230 Ultrasonic Cleaner, Coltene/Whaledent Inc., Akron, OH, USA). Subsequently, implants were inserted into cold-polymerized acrylic resin (Meliodent, Kulzer GmbH, Hanau, Germany) covering 30% carbon fiber-reinforced PEEK tubes that reflect natural jawbone, meeting ISO 14801 [26] elastic module values (polymethyl methacrylate: 3.3 GPa; spongy bone: 1–5 GPa; PEEK: 8 GPa; cortical bone in 1 mm thickness: 6–20 GPa), up to the first implant grooves, to show the physiological state that ensues due to the bone remodeling in the first year after implantation (approximately 0.5–1 mm bone loss) [27]. The specimens were embedded at a 45° angle labiolingually to mimic the intraoral position of the upper and lower anterior teeth, thus the intraoral forces [28]. After the internal surfaces were sandblasted with 50 μm Al_2_O_3_ at 2 bar air pressure, restorations were cemented to monobloc implant abutments (Korostar plus, BEGO GmbH & Co. KG, Bremen, Germany) as stated by the manufacturer’s instructions. Then, restorations were cleaned with alcohol, dried with compressed air, and cemented using dual-cured adhesive resin cement (Panavia F2.0, Kuraray Co., Ltd., Tokyo, Japan) [29]. All of the specimens were stored at room temperature for 24 h following cementation. Before all experimental procedures were followed, instrumentation calibration and precision of the devices, as well as the repeatability and consistency of the tests, were checked.

### 2.2. Chewing Simulation and Static Loading

**Figure 2:**
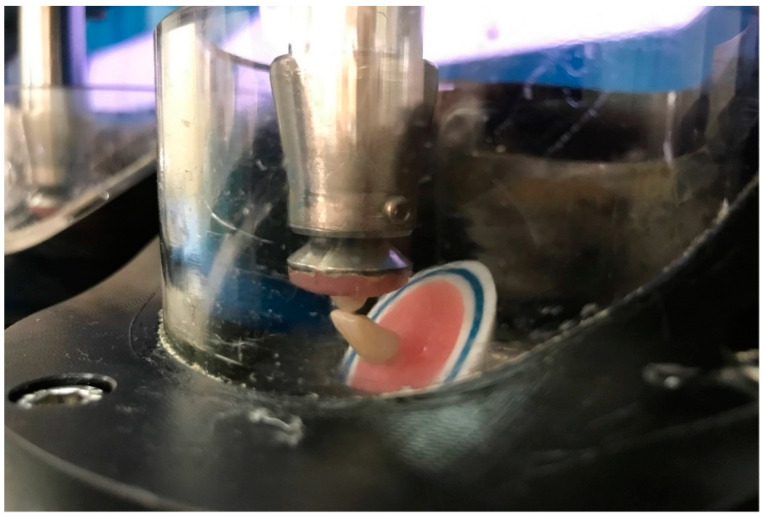
Image of the sample placements and applied loadings in chewing simulation test.

The dynamic loading test used feldspathic ceramic cylindrical balls with a 6 mm diameter as antagonists (IPS Classic V, Ivoclar Vivadent AG, Schaan, Liechtenstein). To determine the maximum contact position of the antagonist, the palatal surface was used for the maxillary region, while for the mandibular region, it was the incisal edge of the restorations. This was accomplished while mimicking intraoral physiological conditions (Figure 2 [28,30]. The chewing simulation’s horizontal movements were adjusted to the labial direction for the samples in the upper anterior position and to the lingual direction for the samples in the lower anterior position. This was accomplished while considering the physiological protrusive movements of the lower jaw [30]. A load of 98 N (10 kg) was applied to the samples through the chewing simulation (MOY-101 Chewing Simulator, ModDental, Esetron Mechatronics Müh. San. Tic. Ltd. Şti., Ankara, Turkey), and the other parameters equal to five years of oral service [19,20,31] are as follows: chewing cycles: 1,200,000; cycle frequency: 1.6 Hz; applied weight per sample: 98 N (10 kg); vertical movement: 6 mm; vertical speed: 60 mm/s; horizontal movement: 0.5 mm; horizontal speed: 50 mm/s; thermal cycles: 6000; cold bath temperature: 5 °C; hot bath temperature: 55 °C; cold–hot dwell time: 60 s. The simulation device has recorded the number of cycles at which the samples failed. In addition, in this study, the “soft contact” mode of the chewing simulator was activated to minimize the initial overloading above desired forces, thus providing fast descending speed [32].

**Figure 3:**
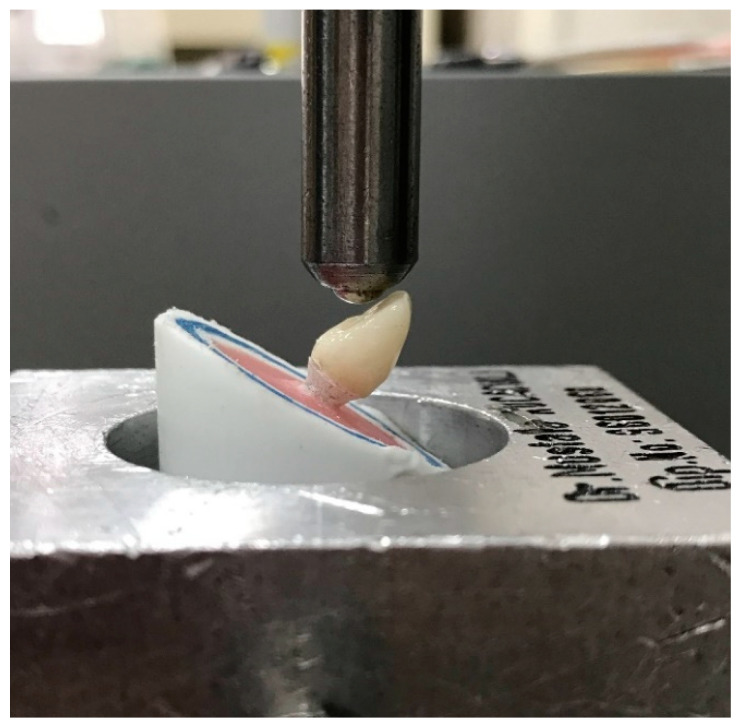
Image of the sample placements and applied loadings in static loading test.

After the chewing simulation, the surviving samples were subjected to a static loading test (Lloyd LRX Universal Testing Device, Lloyd Instruments Ltd., Hampshire, UK) to probe the maximum fracture loads. The identical semicircular steel ball 6 mm in diameter was used to apply static loads in the same directions and points as the chewing simulation test. A 1 mm thick piece of aluminum foil was positioned between the crowns and the steel ball to disseminate the forces evenly. The test was conducted at a speed of 1 mm/min until the load caused failure, and the force required for failure was recorded in Newtons (N) (Figure 3).

After conducting dynamic and static loading tests, the specimens were carefully extracted from the acrylic resin blocks and examined for fracture levels and failure types. Statistical analysis was conducted using SPSS software (version 21). The normality of the data distribution was assessed using the Shapiro–Wilk test. Survival rates of the groups were compared using the Kaplan–Meier log-rank test. The fracture strength values were estimated using nonparametric Kruskal–Wallis and Mann–Whitney U tests. Differences between groups were approximated at a significance level of 0.05.

### 2.3. Raman Spectroscopy Evaluation

**Figure 4:**
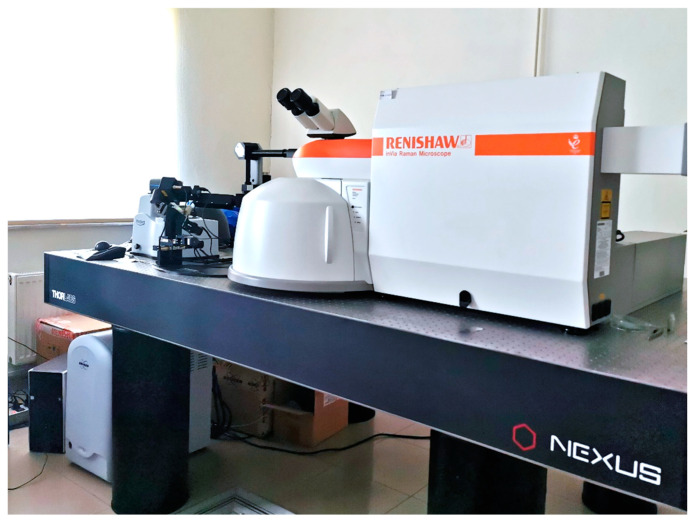
Raman spectroscopy device used in study.

Raman spectroscopy (Renishaw inVia Raman Microscope, İstanbul, Turkey) equipped with a charge-coupled detector (high-resolution charge-coupled device (CCD) camera, 5–20 micron) (Figure 4) was used to determine the phase composition at various points on the surface of the implants (cervical area, thread root, and thread crest) before the chewing simulation. After the dynamic and static loading tests, evidence of phase transformation at the fracture surfaces of the implant bodies was collected. To map the phase fractions and stresses, an Ar-ion laser with a wavelength of 633 nm was used with 5% laser power. The spectra were recorded by averaging five successive measurements, each with a 10-s integration time. Three measurements were taken per implant on a relatively flat part of the fracture surfaces.

### 2.4. Mapping of the Phases

The Raman spectra were investigated using integrated areas under the tetragonal and monoclinic peaks to define the monoclinic fraction. These areas were calculated using the linear background method and integration of the peak areas of all spectra. The typical spectrum of monoclinic zirconia displays 18 typical bands. The bands at 180/190 cm^−1^ and 476 cm^−1^ are highly intense and therefore distinct. Only six bands are visible in the Raman spectrum of tetragonal zirconia compared to the monoclinic phase. The two bands at 259 cm^−1^ and 642 cm^−1^ are both noteworthy. Cubic zirconia shows only one broadband at around 600 cm^−1^, which has a high background signal. Different zirconia phases can be easily discerned by their unique Raman bands [33,34,35].

### 2.5. Micro-CT Evaluation

**Figure 5:**
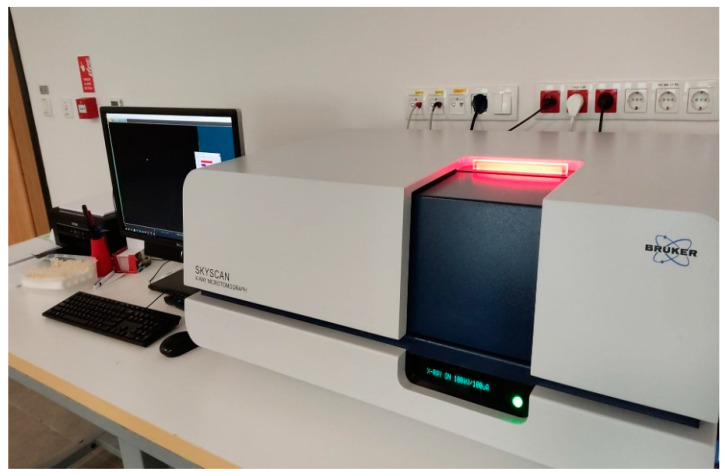
Micro-CT device used in study.

After the Raman spectroscopy evaluations, the fractured parts of each sample were precisely aligned and bonded for micro-CT measurements. A desktop micro-CT system (Bruker Skyscan 1275, Kontich, Belgium) (Figure 5) was utilized to scan the fracture areas of the samples. The scanning conditions were as follows: 100 kVp, 100 mA beam current, 1 mm Cu filter, 10.1 µm pixel size, and rotation at 0.5 steps. To underestimate ring artifacts, air calibration of the detector was performed before each scan. The object under examination was rotated completely around its axis, making a 360-degree turn, and this process was completed within a time frame of 5 min. The mean scanning time was around 1 h. The beam-hardening correction was performed, and optimal contrast limits were set based on prior scanning and reconstruction of the implants, following the manufacturer’s instructions.

### 2.6. Micro-CT Image Analysis

**Figure 6:**
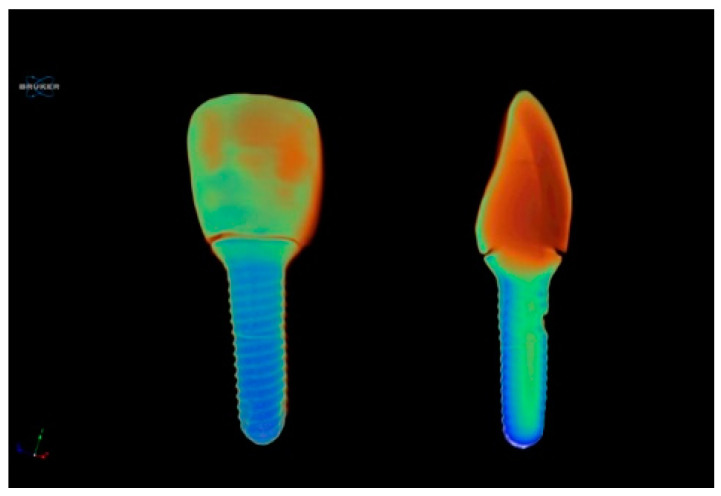
The frontal and proximal images of the specimens that subjected to micro-CT analysis. The fracture lines and the fractured parts which are precisely aligned and bonded can be seen.

The NRecon software (ver. 1.7.4.2, SkyScan, Kontich, Belgium) and CtAn (ver. 1.17.7.2, SkyScan) were utilized for the scanning and digital evaluations of the specimens, using the modified algorithm clarified by Feldkamp et al. [36] to receive axial, two-dimensional, 1000 × 1000-pixel images. The ring artifact correction and smoothing were set at 10, and the beam artifact correction was at 60% to reconstruct the parameters. Contrast limits were used following SkyScan instructions. Using the NRecon software (Skyscan), the images obtained by the scanner were reconstructed to illustrate 2-dimensional slices of the examples. A total of 1014 cross-sectional images were reconstructed from the entire volume. Then, the CTAn (v.1.23.02+, Bruker micro-CT, Kontich, Belgium) and the DataViewer program (v1.5.6.2; Bruker micro-CT) were used to investigate the scans and section the 3D models (Figure 6). All reconstructions were completed using a 21.3-inch flat-panel color-active matrix TFT medical display (NEC MultiSync MD215MG, Munich, Germany) with a resolution of 2048–2560 at 75 Hz and a 0.17 mm dot pitch operated at 11.9 bits. The observers were provided with the capability to adjust the magnification of the sections while being blinded to the study groups.

### 2.7. Gap Measurements

The CTAn software (v.1.23.02+, Bruker micro-CT, Kontich, Belgium) was used to calculate the volume between the fracture sides of the implants. First, the original grayscale images underwent noise reduction using a Gaussian low-pass filter, and an automatic segmentation threshold was applied using CtAn software. A thresholding process (binarization) was used to convert the image to black and white pixels by analyzing the range of gray levels. For each slice, a region of interest was selected to contain a single object fully, facilitating the calculation of volumes. The intersection surface (I.S.) and total porosity were also calculated using CtAn software. The software used for I.S. analysis calculates both the total surface area of the region of interest (ROI) and the area of the ROI that intersects with the binarized area. This parameter represents the contact surface area. With this method, we can measure the contact surface at virtual boundaries of the ROI, which were located at any selected distance from the tissue surface. This provides an extra dimension for studying the contact between different areas. For the total porosity measurement, the density of a material is defined as the ratio between its mass and volume. Therefore, a measure of porosity can be obtained by determining these parameters when the solid density of the bulk material is known. The porosity was calculated as pore volume/specimen volume ×100%. Three-dimensional visualization and qualitative assessment of the samples were conducted using the CTVox software (version 3.3.0.r1403, Bruker micro-CT).

## 3. Results

### 3.1. Dynamic and Static Loading

**Figure 7:**
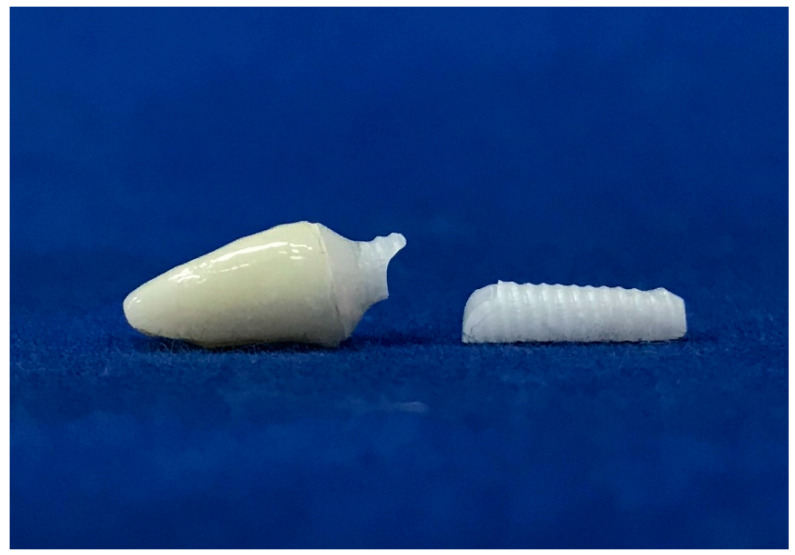
Unstable conical crack propagation has been seen close to the implant neck with a 3.0 mm diameter implants.

**Figure 8:**
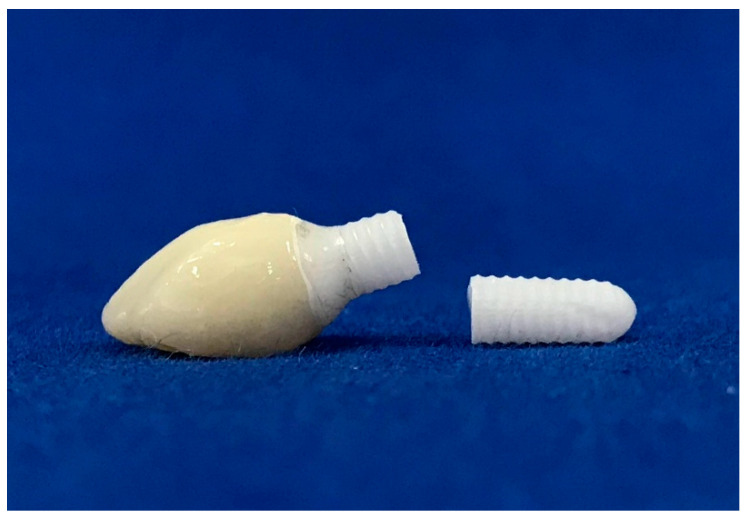
Cone cracks have been seen around the fourth and fifth threads of the 3.7 mm diameter implants which have failed under static loading.

**Figure 9:**
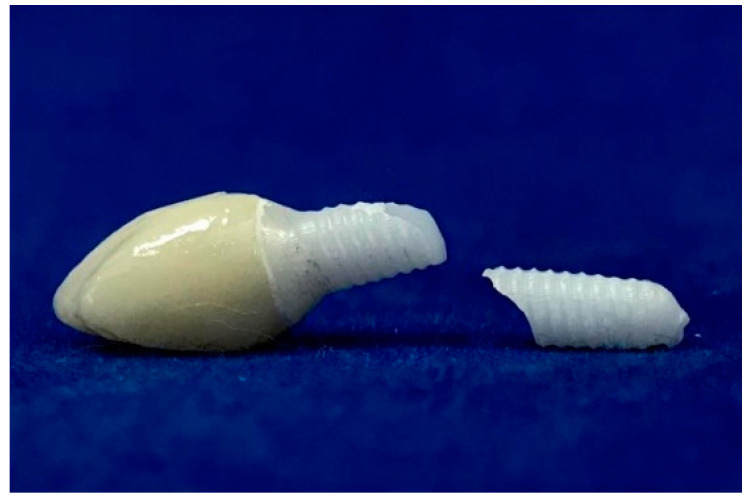
Lateral cracks have been occurred around the fourth and fifth threads of the 3.7 mm diameter implants which have failed under dynamic loading.

The survival rates of the groups and failure cycles of the specimens during the chewing simulation are exhibited in Table 2. Accordingly, the Kaplan–Meier log-rank test results revealed statistically significant differences in survival rates between groups. After subjecting the samples to static loading tests, the differences between the groups that had survived the chewing simulation were found to be insignificant (Table 3). All fractures during dynamic and static loading tests were implant-body fractures with levels varying by implant diameter. Cone, median, and half-penny cracks in unstable propagation were observed close to the implant neck with a 3.0 mm diameter (Figure 7). Lateral cracks occurred in 3.7 mm diameter implants that failed under dynamic loading (Figure 8), although cone cracks were observed in 3.7 mm diameter implants that failed under static loading. All fractures were observed around the fourth and fifth threads of the implants with a diameter of 3.7 mm regardless of whether they failed under dynamic or static loading (Figure 9).

### 3.2. Raman Spectroscopy

**Figure 10:**
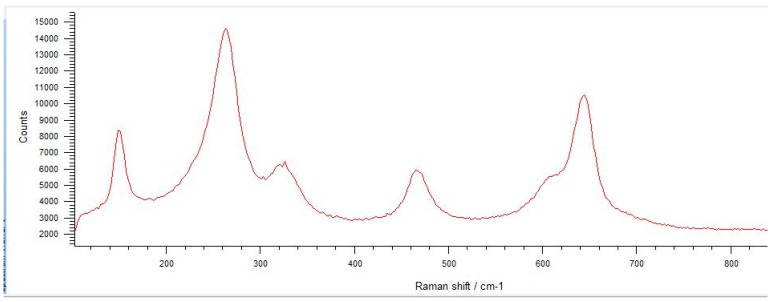
It can be seen the Raman bands indicate the tetragonal phase in the spectra obtained from the untreated samples. Only six bands are visible in the Raman spectrum of tetragonal zirconia. The two bands at 259 cm^−1^ and 642 cm^−1^ are both noteworthy.

**Figure 11:**
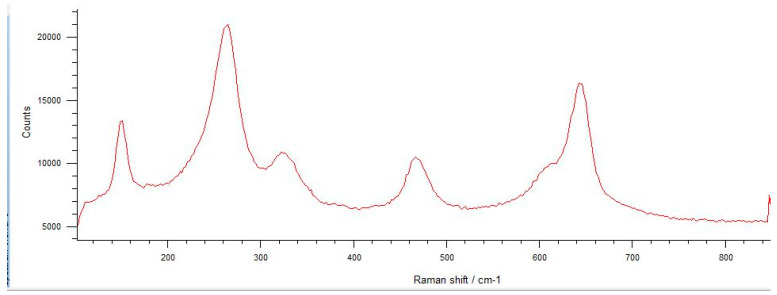
The spectra obtained from the fracture surfaces of 3.0 mm implants, all of which have failed early under dynamic loading. Raman bands indicating the tetragonal phase were seen according to two bands at 259 cm^−1^ and 642 cm^−1^, which are both noteworthy, and no phase transformation was seen.

**Figure 12:**
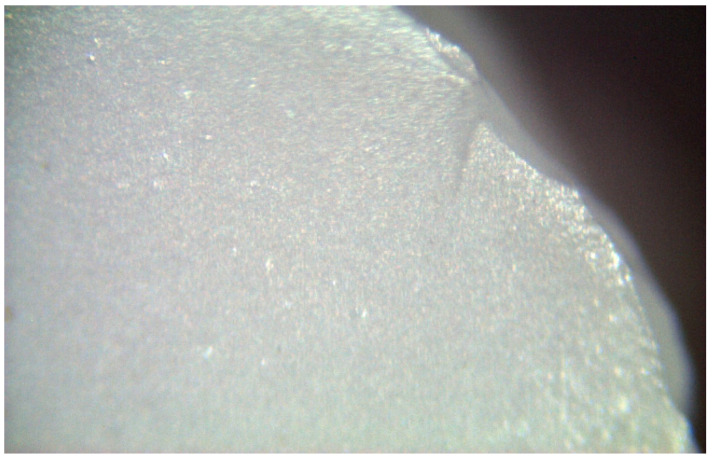
Fracture surfaces of 3.0 mm implants was smooth, and cone microcracks were observed.

**Figure 13:**
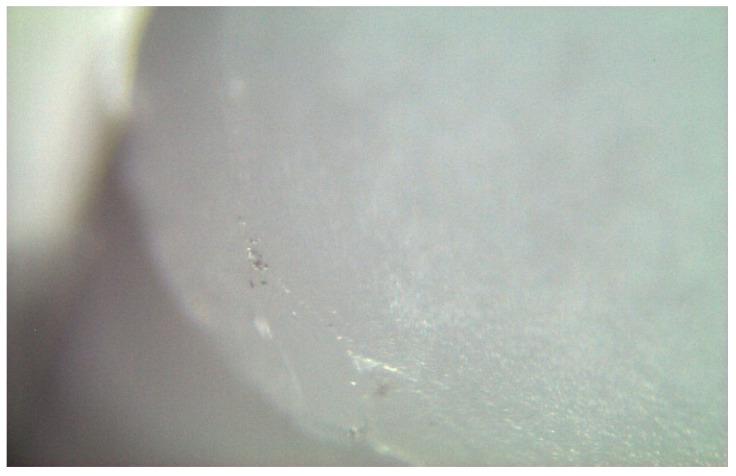
Irregular crack propagation (median) on the smooth fracture surface of the 3.0 mm implants.

**Figure 14:**
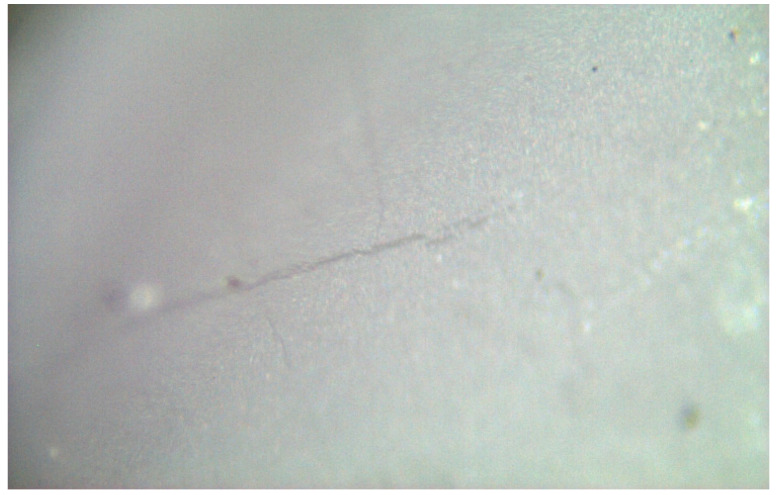
Irregular crack propagation (half-penny) on the smooth fracture surface of the 3.0 mm implants.

**Figure 15:**
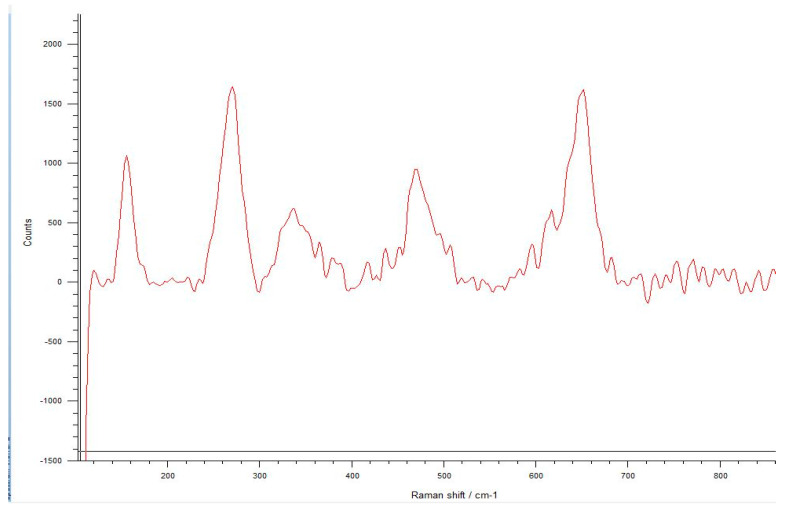
In the Raman spectra measured from the microcracks located close to the fracture area of the 3.0 mm implants, phase transformation is observed. The typical spectrum of monoclinic zirconia has 18 characteristic bands, and compared to the tetragonal phase, the bands at 180/190 cm^−1^ and 476 cm^−1^ are more distinct because of the high intensity.

The spectra obtained from the untreated samples before mechanical tests (Figure 10) were similar to those obtained from the fracture surfaces of 3.0 mm implants (Figure 11), all of which failed early under dynamic loading. Raman bands indicating the tetragonal phase were observed, and no phase transformation was observed; however, microcracks were observed and the fracture surface was smooth (Figure 12). In the spectra measured from the median and half-penny microcracks located close to the smooth fracture surface of the 3.0 mm implants (Figure 13 and Figure 14), a phase transformation was observed (Figure 15).

**Figure 16:**
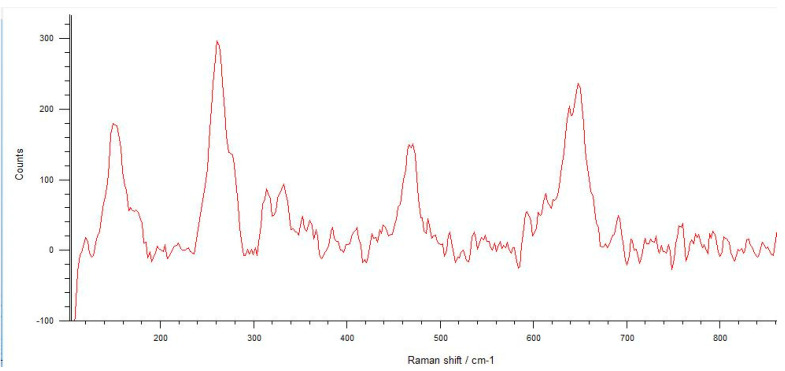
Phase transformation with the bands at 180/190 cm^−1^ and 476 cm^−1^ is more distinct because of the high intensity seen in the Raman spectra observed from 3.7 mm diameter implants that were fractured during chewing simulation.

**Figure 17:**
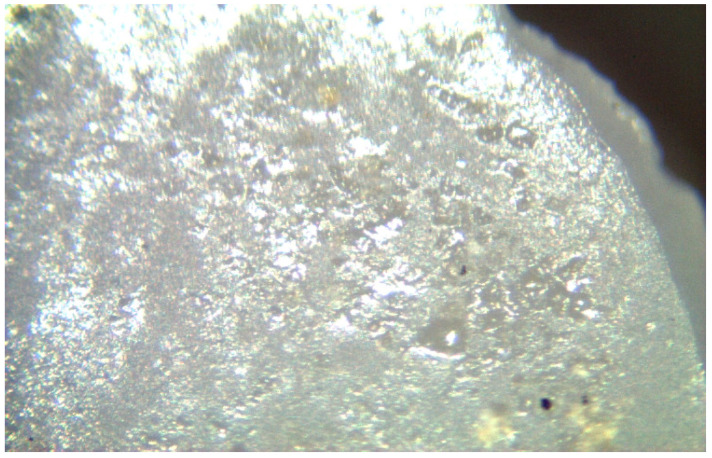
The fracture surface was porous in 3.7 mm diameter implants, which were fractured during chewing simulation.

**Figure 18:**
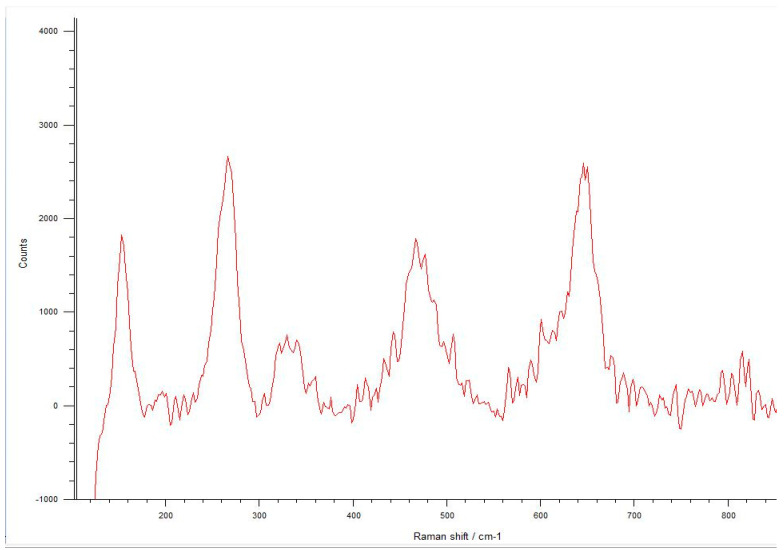
Spectra of phase transformation are observed in 3.7 mm diameter implants that were fractured under static loading, indicating that monoclinic and tetragonal phases coexist with the two important bands at 259 cm^−1^ and 642 cm^−1^ for the tetragonal phase and the bands at 180/190 cm^−1^ and 476 cm^−1^ for the monoclinic phases.

**Figure 19:**
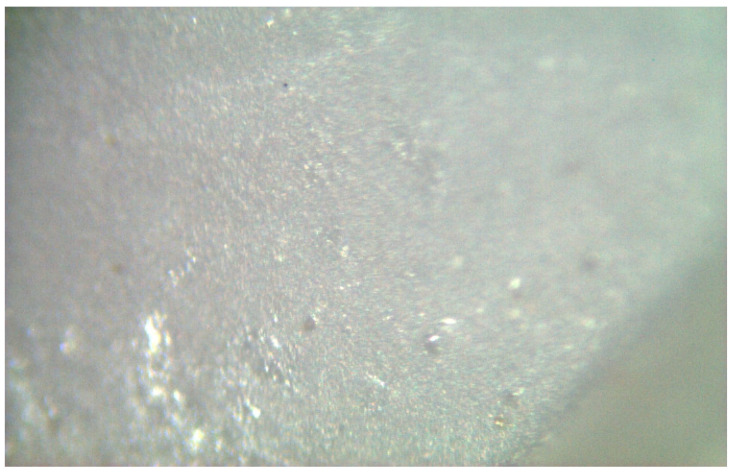
A less porous fracture surface was observed in the 3.7 mm diameter implants that were failed under static loading.

In implants with a diameter of 3.7 mm, which were fractured during chewing simulation, phase transformation was clearly observed in the Raman spectra (Figure 16). In addition, a straight fracture line was followed near the implant body, and the fracture surface was porous (Figure 17). In implants that were fractured under static loading, phase transformation spectra were observed, indicating that monoclinic and tetragonal phases coexisted (Figure 18), and an irregular fracture line and a less porous fracture surface were observed in the implant body (Figure 19). In addition to the Raman measurement results of 3.7 mm implants, no difference was found in the fracture types due to different crown restorations.

### 3.3. Micro-CT

According to the results, the median, minimum, and maximum values of the groups’ gap volume, intersection surface, total porosity, and implant fracture volume values are shown in Table 4. Micro-CT analysis showed that the implant-fracture volume values could be measured in one sample for Group 11-S, in three samples for Group 11-A, in all samples for Groups 31-S and 31-A, and in two samples for Group 13-A. In contrast, for Group 13-S, no implant-fracture volume values were observed in any of the samples. The total porosity values were lower for Groups 31-S and 31-A than for the other groups, and the implant-fracture volume values were higher than for the other groups.

## 4. Discussion

According to the results of this study, narrow-diameter implants and implants with angled abutments have lower fatigue and fracture strength, as reported in previous studies [37,38,39,40]. The lower force value in the chewing simulation resulted in failures due to the effect of dynamic loads on the horizontal axis and the thermal cycle. Zirconia is a material that can withstand compressive stresses but is susceptible to shear and tensile stresses. The zirconia implants were exposed to more shear and tensile stresses due to dynamic loads on the horizontal axis. Additionally, thermal cycling has resulted in a decrease in the material’s ability to resist fatigue and withstand fractures. Low-temperature degradation and t→m phase transformations occur in the presence of moisture, temperature, and stress, which may explain these situations [41]. In a previous study, it was found that zirconia implants have enough strength to resist breaking and are significantly affected by aging. However, the authors reported that the presence of microcracks caused by aging did not decrease the implants’ mechanical strength [18,42].

In the literature, the fractures have commonly been observed along the implant body in the threaded area [43,44,45]. Additionally, most fatigue failures were also notified as implant body fractures [17,46]. Decreased implant diameter causes increased stress at the implant neck [38,47], and it has been reported that angled abutments may cause higher stresses in the cervical region [37,47]. In zirconia implants with a 3.25 mm diameter, the initial crack begins at the first turn of the thread in clinical fractures [48]. During an in-vitro study focused on a type of dental implant made from one-piece zirconia, researchers identified two different patterns of fractures that occurred [49]. One of the fracture lines followed a horizontal path along the border where the implant was embedded in the acrylic resin, while the others were either oblique or parallel to the long axis of the implant. In this study, all failures were implant body fractures; no restorations were decemented nor abutment fractures, and there were no visible deformations observed. The type and location of fractures varied based on implant diameter and abutment angle, as in previous studies, which have shown that narrow-diameter and angled implants were irregularly and obliquely fractured near the cervical region [50]. During the chewing simulation and static loading, different fracture types occurred. It has been observed that the fracture line is irregular unstable propagation to a cone geometry [51] in static loading (Figure 9) where the failures occur due to loads above the fracture strength. This may be due to the change in direction of crack propagation due to transformation toughening. The smooth structure of the fracture surface and the absence of a marked phase transformation in the Raman measurements (Figure 18 and Figure 19) can be thought to indicate that these fractures are not due to fatigue strength. The Raman spectra of zirconia’s most significant phases, including the effects of pressure, temperature, grain size, and dopants on the band position, are essential to study. The typical spectrum of monoclinic zirconia has 18 characteristic bands. Among them, the bands at 180/190 cm^−1^ and 476 cm^−1^ are particularly distinct because of their high intensity. Only six bands are visible in the Raman spectrum of tetragonal zirconia compared to the monoclinic phase. The two most significant bands for identification purposes are those at 259 cm^−1^ and 642 cm^−1^. Cubic zirconia, on the other hand, has only one broad band around 600 cm^−1^ with a high background. It is possible to easily distinguish the Raman bands of different zirconia phases from each other [35,52]. The fact that 3.0 mm implants have no phase transformation in the fracture surfaces in Raman measurements, the fracture surfaces are smooth, and they have irregular fracture lines and microcracks as a result of Raman and micro-CT analysis, indicate transformation toughening that shows that the failures in dynamic loading occur due to exposure to loads above the fracture strength, not due to fatigue strength (Figure 11, Figure 13 and Figure 14, Table 4). The failure of 3.7 mm implants in dynamic loading is thought to be due to fatigue, since a marked phase transformation is observed in Raman measurements as indicated by the LTD, the fracture surfaces were porous, and a straight fracture line is observed. (Figure 16 and Figure 17, Table 4). It has been observed that the failure line of 3.7 mm implants under static loading is irregular conical cracks in static loading, where damage occurs due to loads above the fracture strength. This may be because the crack propagation direction has changed due to transformation hardening. The relatively smooth structure of the fracture surface and the absence of a marked phase transformation in Raman measurements can be considered as an indication that these fractures are not due to fatigue failure. In addition, the fact that fracture occurs in static loading under a lower force than in dynamic loading suggests that the phase transformation (monoclinic and tetragonal phases appear together) that initiates in dynamic loadings reduces the fracture strength [53] (Figure 18 and Figure 19).

It can be said that microcracks located close to the surface on the fracture surface of the 3.0 mm implant confirm the transformation toughening (Figure 13, Figure 14 and Figure 15). When zirconia crystals are subjected to stress, they undergo a phase transformation called “phase transformation toughening” and significantly increase the fracture strength of the material, much higher than the other dental ceramics, by the crack-inhibiting effect. This transformation from tetragonal to monoclinic is a one-way process (at room temperature), which means after the t–m transformation has occurred, zirconia cannot show the transformation toughening. Nevertheless, LTD, or aging, occurs when tetragonal crystals transform on the surface slowly into monoclinic crystals accelerated by moisture, vapor, temperature, mechanical stress, grain size, the distribution of stabilizing oxides, the surface, and manufacturing defects. In this case, the mechanical properties of the material decrease and the risk of spontaneous catastrophic failure increases [54,55]. Although the mechanical properties of cyclically loaded implants were decreased, aging was not considered responsible, and the zirconia implants before and after cyclic loading were found to be acceptable for clinical applications [17]. However, as previously stated, t-m transformation to a specific degree can enhance the mechanical properties of Y-TZP, but there is a narrow range between the improvement and destruction of mechanical properties of Y-TZP, as further aging will lead to property deterioration [56,57]. Within the limitations of the research, experimental implants with narrow diameters and angled abutments were produced from 3Y-TZP material. It is necessary to investigate the zirconia-based materials and develop the stabilization oxides to ensure that transformation toughening supports the mechanical properties and to avoid LTD at a level that will cause the mechanical properties to deteriorate.

In a previous study in which the stress level around the implant and bone was measured by finite element analysis under oblique loadings of 2.9 mm diameter monoblock zirconia and titanium implants, it was shown that zirconia implants caused fewer stress levels in the bone compared to titanium implants, and there were also similar stress levels in the bodies of the titanium and zirconia implants [58]. However, from the results obtained within the limitations of the study, a 3.0 mm implant diameter is not appropriate for clinical use because of the lower survival rates. The fracture strengths of zirconia implants with a 3.7 mm diameter are above the maximum physiological intraoral forces and have sufficient mechanical strength to be used in clinical applications, while the fatigue resistances were nominal, and it may cause some mechanical complications in the long-term clinical follow-up. For this reason, a 3.7 mm implant diameter can be considered a critical diameter for monobloc zirconia implants. Therefore, further studies are required to validate reduced diameter zirconia implants as a reliable alternative.

## 5. Conclusions

Zirconia has made a rapid and remarkable entry and acceleration in the field of dental implant application. In preclinical and clinical studies, zirconia has demonstrated similar results to titanium as an implant material; however, it is necessary not to ignore the aging phenomenon of zirconia, known as low-temperature degradation, in cases of long-term intraoral use. According to the results of this study, phase transformation affects the fracture type and the roughness of the fractured surfaces. While phase transformation has directed microcrack propagation in a way that improves fracture toughness up to a certain level, it has caused destructive effects after a certain level. While zirconia implants have biological advantages, they have gained popularity as an alternative to titanium due to their aesthetic advantages. However, their fatigue strength is a concern for clinical applications in the anterior region, where narrow diameters and angled abutments are necessary. In this field, in vitro studies are needed on the development of narrow-diameter zirconia implants.

## Figures and Tables

**Table 1:** All details of sample groups.

Groups	Crown Forms	Number	Abutment Angle	Implant Diameter	Implant Length
11-S	Upper incisor	8	Straight abutment	3.7 mm	11.5 mm
11-A	Upper incisor	8	15° angled abutment	3.7 mm	11.5 mm
31-S	Lower incisor	8	Straight abutment	3.0 mm	11.5 mm
31-A	Lower incisor	8	15° angled abutment	3.0 mm	11.5 mm
13-S	Upper canine	8	Straight abutment	3.7 mm	11.5 mm
13-A	Upper canine	8	15° angled abutment	3.7 mm	11.5 mm

**Table 2:** Minimum (min.), maximum (max.), and median (med.) cycles in which the failures occurred, standard deviations (std. dev.), and survival rates of groups after chewing simulation equivalent to five years. (Same superscript lowercase letters in columns represent no statistically significant differences (*p* > 0.05)). Different superscript lowercase letters represent statistically significant differences between columns (*p* < 0.05).

Groups	Med. (Std. Dev.)	Min.	Max.	Total Samples	Failed Samples	Survived Samples	Survival Rates
11-S	1,200,000 (253,710)	578,539	1,200,000	8	1	7	87.5% ^a^
11-A	1,159,823 (472,374)	148,07	1,200,000	8	4	4	50.0% ^b^
31-S	56,296 (48,197)	11,715	100,000	8	8	0	0.0% ^c^
31-A	143 (525.97)	105	1143	8	8	0	0.0% ^c^
13-S	1,200,000 (0)	1,200,000	1,200,000	8	0	8	100.0% ^d^
13-A	1,200,000 (542,235)	139,644	1,200,000	8	2	6	75.0% ^e^

**Table 3:** The minimum, maximum, and median values and standard deviations of the fracture loads. [same superscript lowercase letters in columns represent no statistically significant differences (*p* > 0.05)].

Groups	Median (N) (Standard Deviation)	Minimum (N)	Maximum (N)
11-S	471.63 (77.03) ^a^	332.7	515.77
11-A	316.34 (60.39) ^a^	268.88	388.79
13-S	291.89 (95.28) ^a^	239.48	482.77
13-A	465.07 (157.77) ^a^	306.06	663.94

**Table 4:** According to micro-CT results the median (med.), minimum (min.), and maximum (max.) values of the groups.

Groups	Gap Volume (mm^3^)			Intersection Surface (mm^2^)			Total Porosity (Percent)			Implant Fracture Volume (mm^3^)		
	Med.	Min.	Max.	Med.	Min.	Max.	Med.	Min.	Max.	Med.	Min.	Max.
11-S	37.76	33.46	43.41	3.22	3.17	3.92	94.52	88.6	97.7	4.93	-	-
11-A	42.78	35.74	46.56	3.93	3.06	4.07	81.1	90.33	97	4.68	4.63	5.1
31-S	26.1	23.9	30.56	2.42	2.11	2.65	79.38	70.38	85.16	5.45	4.8	6.66
31-A	34.92	34.5	37.36	3.2	2.15	3.16	78.77	72.29	87.09	5.29	4.45	5.93
13-S	39.5	36.37	40.81	3.41	3.05	3.85	95.74	92.65	98.45	-	-	-
13-A	39.82	37.55	49.74	3.52	3.1	4.09	94.46	85.9	97.12	4.94	4.94	5.03

## Data Availability

The raw data supporting the conclusions of this article will be made available by the authors on request.

## References

[1] Andreiotelli M., Wenz H.J., Kohal R.J. (2009). Are ceramic implants a viable alternative to titanium implants? A systematic literature review. Clin. Oral Implant. Res..

[2] McKinney R.V., Koth D.L. (1982). The single-crystal sapphire endosteal dental implant: Material characteristics and 18-month experimental animal trials. J. Prosthet. Dent..

[3] Sandhaus S. (1968). Technic and instrumentation of the implant CBS (Cristalline Bone Screw). Inf. Odonto-Stomatol..

[4] Depprich R., Zipprich H., Ommerborn M., Naujoks C., Wiesmann H.P., Kiattavorncharoen S., Lauer H.C., Meyer U., Kübler N.R., Handschel J. (2008). Osseointegration of zirconia implants compared with titanium: An in vivo study. Head Face Med..

[5] Piconi C., Maccauro G., Muratori F., Branch del Prever E. (2003). Alumina and zirconia ceramics in joint replacements. J. Appl. Biomater. Biomech..

[6] Pieralli S., Kohal R.J., Jung R.E., Vach K., Spies B.C. (2017). Clinical Outcomes of Zirconia Dental Implants: A Systematic Review. J. Dent. Res..

[7] Scarano A., Di Carlo F., Quaranta M., Piattelli A. (2003). Bone response to zirconia ceramic implants: An experimental study in rabbits. J. Oral Implantol..

[8] Scarano A., Piatelli M., Caputi S., Favero G.A., Piattelli A. (2004). Bacterial adhesion on commercially pure titanium and zirconium oxide disks: An in vivo human study. J. Periodontol..

[9] Wenz H.J., Bartsch J., Wolfart S., Kern M. (2008). Osseointegration and clinical success of zirconia dental implants: A systematic review. Int. J. Prosthod..

[10] Cionca N., Hashim D., Mombelli A. (2017). Zirconia dental implants: Where are we now, and where are we heading?. Periodontology 2000.

[11] Kohal R.J., Weng D., Bachle M., Strub J.R. (2004). Loaded custom-made zirconia and titanium implants show similar osseointegration: An animal experiment. J. Periodontol..

[12] Piconi C., Maccauro G. (1999). Zirconia as a ceramic biomaterial. Biomaterials.

[13] Chevalier J. (2006). What future for zirconia as a biomaterial?. Biomaterials.

[14] Chevalier J., Gremillard L., Deville S. (2007). Low-Temperature Degradation of Zirconia and Implications for Biomedical Implants. Annu. Rev. Mater. Res..

[15] Monzavi M., Zhang F., Meille S., Douillard T., Adrien J., Noumbissi S., Nowzari H., Chevalier J. (2020). Influence of artificial aging on mechanical properties of commercially and non-commercially available zirconia dental implants. J. Mech. Behav. Biomed. Mater..

[16] Pittayachawan P., McDonald A., Young A., Knowles J.C. (2009). Flexural strength, fatigue life, and stress-induced phase transformation study of Y-TZP dental ceramic. J. Biomed. Mater. Res. Part B Appl. Biomater..

[17] Zhang F., Zur Heide C.M., Chevalier J., Vleugels J., Van Meerbeek B., Wesemann C., Dos Santos B.C., Sergo V., Kohal R.J., Adolfsson E. (2020). Reliability of an injection-moulded two-piece zirconia implant with PEKK abutment after long-term thermo-mechanical loading. J. Mech. Behav. Biomed. Mater..

[18] Sanon C., Chevalier J., Douillard T., Cattani-Lorente M., Scherrer S.S., Gremillard L. (2015). A new testing protocol for zirconia dental implants. Dent. Mater..

[19] Tekbaş Atay M., Oğuz Ahmet B.S., Sayın Özel G. (2016). The impotance of thermal and loading cycles in terms of simulation of the oral cavity. J. Dent. Fac. Atatürk Uni..

[20] Krejci I., Lutz F. (1990). In-vitro test results of the evaluation of dental restoration systems. Correlation with in-vivo results. Schweiz. Monatsschr. Zahnmed..

[21] Clarke D.R., Adar F. (1982). Measurement of the crystallographically transformed zone produced by fracture in ceramics containing tetragonal zirconia. J. Am. Ceram. Soc..

[22] Guldberg R.E., Ballock R.T., Boyan B.D., Duvall C.L., Lin A.S., Nagaraja S., Oest M., Phillips J., Porter B.D., Robertson G. (2003). Analyzing bone, blood vessels, and biomaterials with microcomputed tomography. IEEE Eng. Med. Biol. Mag..

[23] Guldberg R.E., Lin A.S., Coleman R., Robertson G., Duvall C. (2004). Microcomputed tomography imaging of skeletal development and growth. Birth Defects Res. C Embryo Today.

[24] Swain M.V., Xue J. (2009). State of the art of Micro-CT applications in dental research. Int. J. Oral Sci..

[25] Kamel M., Vaidyanathan T.K., Flinton R. (2017). Effect of abutment preparation and fatigue loading in a moist environment on the fracture resistance of the one-piece zirconia dental implant. Int. J. Oral Maxillofac. Implant..

[26] (2017). Dynamic Loading Test for Endosseous Dental Implants.

[27] Enderle J.D., Bronzino D.J. (2012). Introduction to Biomedical Engineering. Biomaterials: Types, Properties, and Their Applications.

[28] Rakosi T., Jonas I., Graber T.M., Rateitschak K.H., Wolf H.F. (1993). Orthodontic Diagnosis (Color Atlas of Dental Medicine).

[29] Gargari M., Gloria F., Napoli E., Pujia A.M. (2010). Zirconia: Cementation of prosthetic restorations. Literature review. Oral Implantol..

[30] Donegan S.J., Knap F.J. (1995). A study of anterior guidance. J Prosthodont..

[31] Delong R., Douglas W.H. (1983). Development of an artificial oral environment for the testing of dental restoratives: Bi-axial force and movement control. J. Dent. Res..

[32] Steiner M., Mitsias M.E., Ludwig K., Kern M. (2009). In vitro evaluation of a mechanical testing chewing simulator. Dent. Mater..

[33] Alzyab B., Perry C.H., Ingel R.P. (1987). High-pressure phase transitions in zirconia and yttria-doped zirconia. J. Am. Ceram. Soc..

[34] Phillippi C.M., Mazdiyasni K.S. (1971). Infrared and Raman spectra of zirconia polymorphs. J. Am. Ceram. Soc..

[35] Colthup N.B., Daly L.H., Wiberley S.E. (1990). Introduction to Infrared and Raman Spectroscopy.

[36] Feldkamp L.A., Goldstein S.A., Parfitt A.M., Jesion G., Kleerekoper M. (1989). The direct examination of three-dimensional bone architecture in vitro by computed tomography. J. Bone Miner. Res..

[37] Brosh T., Pilo R., Sudai D. (1998). The influence of abutment angulation on strains and stresses along the implant/bone interface: Comparison between two experimental techniques. J. Prosthet. Dent..

[38] Scherrer S.S., Mekki M., Crottaz C., Gahlert M., Romelli E., Marger L., Durual S., Vittecoq E. (2019). Translational research on clinically failed zirconia implants. Dent. Mater..

[39] Schiegnitz E., Al-Nawas B. (2018). Narrow-diameter implants: A systematic review and meta-analysis. Clin. Oral Impl. Res..

[40] Shemtov-Yona K., Rittel D., Machtei E.E., Levın L. (2014). Effect of dental implant diameter on fatigue performance. Part II: Failure analysis. Clin. Implant Dent. Relat. Res..

[41] Jing Z., Ke Z., Yihong L., Zhijian S. (2014). Effect of multistep processing technique on the formation of microdefects and residual stresses in zirconia dental restorations. J. Prosthodont..

[42] Kammermeier A., Rosentritt M., Behr M., Schneider-Feyrer S., Preis V. (2016). In vitro performance of one- and two-piece zirconia implant systems for anterior application. J. Dent..

[43] Attard L., Lee V., Le J., Lowe C., Singh V., Zhao J., Sharma D. (2022). Mechanical Factors Implicated in Zirconia Implant Fracture Placed within the Anterior Region—A Systematic Review. Dent. J..

[44] Chrcanovic B.R., Kisch J., Albrektsson T., Wennerberg A. (2018). Factors influencing the fracture of dental implants. Clin. Implant Dent. Relat. Res..

[45] Li Y., Yu H.J., Qiu L.X. (2022). Clinical classification and treatment decision of implant fracture. Beijing Da Xue Xue Bao Yi Xue Ban.

[46] Holanda K.A.B., Caldas R.A., Amaral M., Vitti R.P. (2021). Biomechanical evaluation of anterior implants associated with titanium and zirconia abutments and monotype zirconia implants. J. Prosthodont. Res..

[47] Andreiotelli M., Kohal R.J. (2009). Fracture strength of zirconia implants after artificial aging. Clin. Implant Dent. Relat. Res..

[48] Gahlert M., Burtscher D., Grunert I., Kniha H., Steinhauser E. (2012). Failure analysis of fractured dental zirconia implants. Clin. Oral Implant. Res..

[49] Kohal R.J., Wolkewitz M., Tsakona A. (2011). The effects of cyclic loading and preparation on the fracture strength of zirconium-dioxide implants: An in vitro investigation. Clin. Oral Implant. Res..

[50] Burkhardt F., Harlass M., Adolfsson E., Vach K., Spies B.C., Kohal R.-J. (2021). A Novel Zirconia-Based Composite Presents an Aging Resistant Material for Narrow-Diameter Ceramic Implants. Materials.

[51] Cook R.F., Pharr G.M. (1990). Direct observation and analysis of indentation cracking in glasses and ceramics. J. Am. Ceram. Soc..

[52] Schrader B. (2008). Infrared and Raman Spectroscopy: Methods and Applications.

[53] Kim W., Song E., Ju K., Lim D., Han D., Jung T., Jeong Y., Lee J.-H., Kim B. (2020). Mechanical assessment of fatigue characteristics between single-and multi-directional cyclic loading modes on a dental implant system. Materials.

[54] Camposilvan E., Leone R., Gremillard L., Sorrentino R., Zarone F., Ferrari M., Chevalier J. (2018). Aging resistance, mechanical properties and translucency of different yttria-stabilized zirconia ceramics for monolithic dental crown applications. Dent. Mater..

[55] Lughi V., Sergo V. (2010). Low temperature degradation -aging- of zirconia: A critical review of the relevant aspects in dentistry. Dent. Mater..

[56] Lawson S. (1995). Environmental degradation of zirconia ceramics. J. Eur. Ceram. Soc..

[57] Osman R.B., Swain M.V. (2015). A critical review of dental implant materials with an emphasis on titanium versus zirconia. Materials.

[58] Al-Zordk W., Ghazy M., El-Anwar M. (2020). Stress analysis around reduced-diameter zirconia and titanium one-piece ımplants with and without microthreads in the neck: Experimental and finite element analysis. Int. J. Oral Maxillofac. Implant..

